# Later-age neutering causes lower risk of early‐onset urinary incontinence than early neutering–a VetCompass target trial emulation study

**DOI:** 10.1371/journal.pone.0305526

**Published:** 2024-07-03

**Authors:** Camilla Pegram, Karla Diaz-Ordaz, Dave C. Brodbelt, Yu-Mei Chang, Jon L. Hall, David B. Church, Dan G. O’Neill

**Affiliations:** 1 Pathobiology and Population Sciences, The Royal Veterinary College, Hawkshead Lane, North Mymms, Hatfield, Herts, United Kingdom; 2 Department of Statistical Science, University College London, London, United Kingdom; 3 Research Support Office, The Royal Veterinary College, Hatfield, Herts, United Kingdom; 4 School of Veterinary Medicine and Science, University of Nottingham, Nottingham, Sutton Bonington, United Kingdom; 5 Clinical Science and Services, The Royal Veterinary College, Hawkshead Lane, North Mymms, Hatfield, Herts, United Kingdom; University of Life Sciences in Lublin, POLAND

## Abstract

There is growing evidence supporting clinically important associations between age at neutering in bitches and subsequent urinary incontinence (UI), although much of this evidence to date is considered weak. Target trial emulation is an innovative approach in causal inference that has gained substantial attention in recent years, aiming to simulate a hypothetical randomised controlled trial by leveraging observational data. Using anonymised veterinary clinical data from the VetCompass Programme, this study applied the target trial emulation framework to determine whether later-age neutering (≥ 7 to ≤ 18 months) causes decreased odds of early-onset UI (diagnosed < 8.5 years) compared to early-age neutering (3 to < 7 months). The study included bitches in the VetCompass database born from January 1, 2010, to December 31, 2012, and neutered between 3 and 18 months old. Bitches were retrospectively confirmed from the electronic health records as neutered early or later. The primary outcome was a diagnosis of early-onset UI. Informed from a directed acyclic graph, data on the following covariates were extracted: breed, insurance status, co-morbidities and veterinary group. Inverse probability of treatment weighting was used to adjust for confounding, with inverse probability of censoring weighting accounting for censored bitches. The emulated trial included 612 early-age neutered bitches and 888 later-age neutered bitches. A pooled logistic regression outcome model identified bitches neutered later at 0.80 times the odds (95% CI 0.54 to 0.97) of early-onset UI compared with bitches neutered early. The findings show that later-age neutering causes reduced odds of early-onset UI diagnosis compared with early-age neutering. Decision-making on the age of neutering should be carefully considered, with preference given to delaying neutering until after 7 months of age unless other major reasons justify earlier surgery. The study is one of the first to demonstrate successful application of the target trial framework to veterinary observational data.

## Introduction

Surgical neutering of bitches is generally viewed as an important means of population control, that offers both health-related and behavioural benefits, including prevention of mammary tumours, pyometra and problem behaviours associated with oestrus or pseudopregnancy [1]. However, neutering has also been reported as associated with negative health-related effects in bitches, for example, the development of joint disease, neoplasia and urinary incontinence (UI) [1–3]. Given an estimated 75% of bitches in the UK will be neutered during their lifetime [4], the associated risks from neuter decision-making are magnified at a population level. Evidence for the optimal age to neuter bitches remains inconsistent [5], although it has been suggested this decision should be made on a bitch-by-bitch basis, in line with the breed-specific effects for age of neutering [6].

UI is defined as the involuntary loss of urine during the filling phase of the bladder [7] and is a common canine condition, with overall prevalence in neutered and entire bitches under primary-care practice in England estimated at 3.14% [8]. The most common cause of UI in puppies (< 6 months) is ureteral ectopia [9, 10], whilst in adult dogs, urethral sphincter mechanism incompetence (USMI) is the most common, with neutered females at particular risk [3, 8, 11, 12]. In bitches, clinical history and pattern recognition often lead to a presumptive diagnosis of UI, although a recent ACVIM consensus statement has provided a visual aid for the recommended diagnosis and management pathway for UI in dogs [13].

Neutering, breed, body size, tail-docking, obesity and age have all previously been reported as risk factors for UI [3, 8, 12, 14, 15]. Anatomic abnormalities have also been reported in affected bitches, including shorter urethras and more caudally positioned bladder necks [14]. Previous studies have reported overwhelming evidence that neutered bitches are at increased risk of UI [3, 8, 11, 12, 16, 17]. From a pathophysiological perspective, the associations between neutering and UI are not fully clear, but neurological, vascular and hormonal changes have been proposed [12, 14].

In addition to the overwhelming evidence for an association between neutering per se and UI, there is also growing evidence supporting an association between the age at neutering and a subsequent UI diagnosis although the strength of this evidence is still weak [3, 18, 19]. A systematic review in 2012 reported some weak evidence that the risk of UI in bitches decreases as the age at neutering increases, up until 12 months of age, after which the evidence did not support any remaining effect [19]. In that review, a US-based study of shelter dogs reported a higher incidence of UI in bitches that were neutered before 3 months of age compared to after [18]. A more recent study based on UK veterinary primary-care data identified an increased hazard of early-onset UI (first diagnosed at < 8.5 years) within the first 2 years following neutering in bitches neutered before 6 months, compared to bitches neutered at 6 to <12 months [3]. By contrast, two UK questionnaire-based studies of bitches under primary veterinary care did not find a significant association between age at neutering and UI [15, 17]. It was suggested this difference may be explained, in part, by the typically different ages at neutering in the UK compared to the US [19]. A previous UK-based study reported 22.1% of dogs are neutered by 7 months of age compared to 59.9% by 15 months [20]. Although there is no direct comparator for the US currently available, it has been suggested neutering dogs by 6 months of age is standard practice in the US [21].

Although the age at neutering may be correlated with whether bitches are neutered before or after their first oestrus, any associations between the timing of neutering relative to the first oestrus and later development of UI in the bitch remain unclear [19]. A UK cohort study of bitches under primary veterinary care reported evidence of an increased risk of acquired UI in bitches neutered before, rather than after, their first oestrus [17]. However, another study identified no significant association between the timing of neuter relative to onset of oestrus and UI [22]. A UK case-control study based on veterinary primary-care data considered evaluating the association between timing of neuter relative to onset of oestrus and UI but data on the timing of neuter relative to first oestrus were missing in 76.9% of bitches in the study, and therefore this analysis was not carried out [12].

The traditional gold standard study design using causal inference to estimate treatment effects is the randomised controlled trial (RCT), however, RCTs are not always feasible or ethical [23, 24]. Target trial emulation is an innovative approach in causal inference that has gained substantial attention in recent years, aiming to simulate a hypothetical RCT by leveraging observational data [25]. Thus within the target trial framework, an observational study is considered as a conditionally randomised experiment, taking in to account the measured covariates [26, 27]. Successful application of this approach within the veterinary literature has recently been demonstrated, with studies using primary-care EHR data evaluating: 1) whether antimicrobial or gastrointestinal nutraceutical treatment in acute, uncomplicated canine diarrhoea causes improved clinical outcomes [28] and 2) whether surgical or non-surgical management for cranial cruciate ligament rupture in dogs causes improved clinical outcomes [27].

UI has been reported as a major contra-indication for neutering bitches by veterinarians [29]. However, associations between neutering and UI are multifaceted and nuanced. The age at neutering may affect the overall UI risk, although the evidence is currently weak [3, 8]. Additionally, previous studies that attempted to explore the effects of age at neutering on UI diagnosis were limited to reporting associations rather than causal effects [3, 12, 15, 17–19]. Therefore, a fuller evaluation of the causal role of age at neutering in the development of UI is warranted, with recent advances in causal inference methodology now enabling research to progress from studying association to reporting causation [27, 28].

Using anonymised veterinary clinical data from the VetCompass Programme [30], the current study aimed to apply the target trial framework on veterinary primary care observational clinical data to specifically compare the causal effects between neutering at 3 to < 7 months and neutering at ≥ 7 to ≤ 18 months on subsequent diagnosis with early-onset UI in bitches. It was hypothesized that neutering at ≥ 7 to ≤ 18 months causes a decreased odds of early-onset UI diagnosis compared with neutering at 3 to < 7 months [3, 18, 19]. Target trial emulation and causal inference analyses were adopted to attenuate as many sources of bias as possible from the use of observational data in the study [25, 27, 28].

## Materials and methods

### Data source and sample size calculation

VetCompass collates de-identified electronic health record (EHR) data from primary-care veterinary practices in the UK for epidemiological research. Access to the VetCompass database for a specific project requires prior ethical approval, completion of data confidentiality agreement and a stated plan for how the study aims to achieve animal welfare gain. The research data are accessed through a web-based platform (www.vetcompass.org) [30]. The study population included a cohort of bitches born from January 1, 2010 to December 31, 2012, with at least one EHR within VetCompass recorded before 12 months of age, and that were neutered between 3 and 18 months old. UI cases and controls were sampled from the underlying cohort. The target trial approach has mostly been applied to cohort studies, but it can be readily extended to case-control studies when information on desired treatments or confounders is unavailable for the entire cohort but can be obtained for a smaller subset of cases and controls [31]. Available data fields for the current study included a unique animal identifier along with species, breed, date of birth, sex, neuter status, insurance status, clinical information from free-form text clinical notes, bodyweight and treatment with relevant dates.

The study design applied the target trial emulation method [25], similar to a previous studiesbased on primary-care EHR data [27, 28]. Because manual extraction of data from EHRs is time intensive, the case-control design allowed for confounders to be extracted for a requisite subset of cases and controls. Sample size calculations estimated that approximately 734 early-onset UI cases and 734 controls were required to identify if bitches neutered later (defined as ≥ 7 to ≤ 18 months) caused 0.70 times the odds of early-onset UI diagnosis compared with bitches neutered early (defined as 3 to < 7 months) [18], assuming 80% power, 95% confidence interval (CI), a 1:1 ratio of early-onset UI cases to controls and, based on prior evidence, that 80% controls are neutered later [3, 20, 32]. Ethics approval was obtained from the RVC Social Science Ethical Review Board (reference number SR2018-1652).

### Case definition, case finding and covariates

The case definition for early-onset UI required a final diagnosis of UI before 8.5 years of age [3]. In line with VetCompass methods used in several previous studies [33–36], candidate UI cases were identified by applying search terms relevant to the diagnosis and management of UI in the clinical notes and treatment fields (including incont*, usmi, urin* leak*, incompet*, propal*, incuri* and urilin). The search findings were merged, and a random subset of these candidate cases had their clinical notes examined manually in detail to identify bitches that met the UI case definition. All non-candidate bitches were classified as non-cases and were available for consideration as controls. The non-candidate bitches were randomly ordered and the EHRs of a random selection were examined manually in detail to ensure there was no evidence of UI [12].

**Fig 1:**
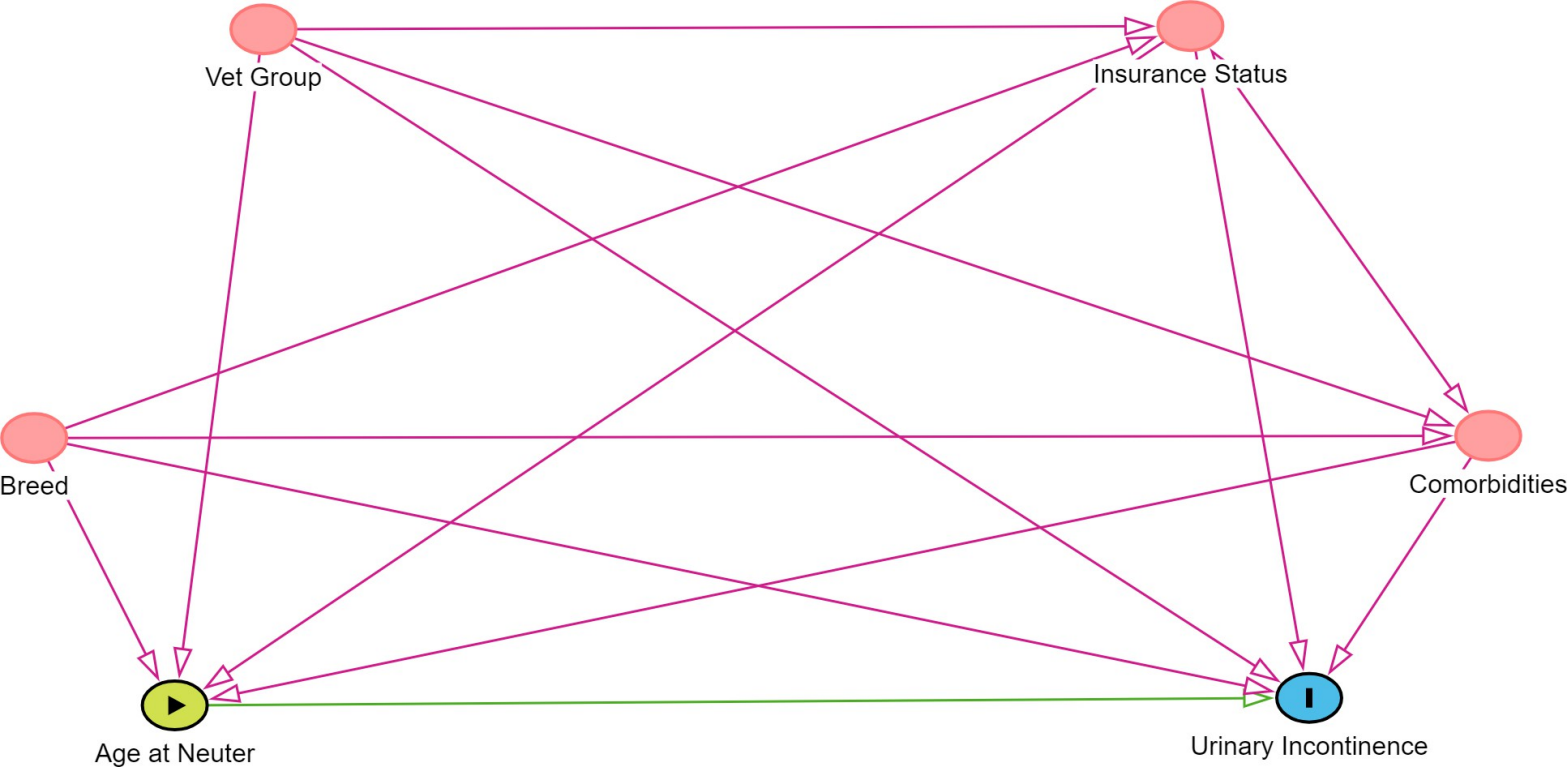
Directed acyclic graph (DAG) based on existing evidence and expert knowledge to estimate beliefs by the research team about the total effect of age at neutering in bitches on early-onset UI diagnosis.

Based on existing published evidence and expert knowledge, a directed acyclic graph (DAG) was constructed using DAGitty version 3.0 that encapsulated prior beliefs by the research team about the causal relationships relevant to the question of interest (Fig 1). The DAG was used to identify which variables should be controlled for in the modelling [37], and therefore required the following data to be additionally extracted: breed, veterinary group, insurance status and chronic comorbidities (Fig 1).

For cases and controls, demographic data were extracted automatically from the VetCompass database, with further data relating to neutering and UI extracted manually from the EHR (Table 1).

**Table 1:** Definition and categorisation of demographic and clinical data extracted from the electronic health records of early-onset UI (< 8.5 years) case and control bitches attending primary-care veterinary practices in the VetCompass™ Programme in the UK (n = 1500).

Data extracted	Definition	Categorisation
Age at neutering	Age at neutering (months).	Early-age neutering (3 to < 7 months) and later-age neutering (≥ 7 to ≤ 18 months). This categorisation was chosen given 22.1% of dogs are neutered by 7 months of age (allowing reasonable comparator groups) and the majority (59.9%) are reported as neutered by 15 months of age [20]. The exact age at neutering (months) was also extracted as a continuous variable for inclusion in the outcome model.
Breed	Breed information entered by the participating practices was cleaned and mapped to a VetCompass breed list derived and extended from the VeNom Coding breed list [38].	Each breed with at least 15 bitches neutered early or later, to support study power, with remaining bitches grouped as either “Purebred–Other” or “Crossbred”.
Insurance status	Status at the final available electronic health record.	“Insured” or “Non-insured”.
Chronic comorbidity	Presence of at least one chronic comorbidity i.e. an ongoing condition of ≥ 3 weeks duration recorded in the electronic health record at 3 months of age. Specific comorbidities with at least 8 bitches affected were included as separate variables.	“Yes” or “No”
Veterinary group	The 5 corporate or partnership practice groups that contributed data to the study.	Categorised as 1–5.

### Target trial specification and emulation

A target trial of interest was specified and emulated using EHR data [25–28, 39]. The protocols of the target trial, and the trial emulation, are summarised in Table 2.

**Table 2:** Specification and emulation of target trial to estimate the causal effect of early compared with later age at neutering on a subsequent outcome of early-onset UI diagnosis.

Protocol Component	Target trial description	Emulated trial using veterinary electronic health records
Research question	What is the causal effect of neutering at 3 to < 7 months versus neutering at ≥ 7 to ≤ 18 months on early-onset urinary incontinence (UI) diagnosis (defined as UI diagnosed at < 8.5 years) in bitches?	Same as target trial.
Eligibility criteria	Bitches born from January 1, 2010 to December 31, 2012, inclusive, and neutered between 3 and 18 months, with at least one electronic health record (EHR) before 12 months of age. An early-onset UI case requires a final diagnosis of UI (or synonym) and/or treatment with either phenylpropanolamine or oestriol. Exclusion criteria comprise: • UI recorded as occurring secondary to a primary neurological condition. • Evidence of congenital UI. • Evidence of urinary tract infection with UI reported to resolve with appropriate treatment of the urinary tract infection. • Evidence that the phenylpropanolamine or oestriol was given for other than UI. Further exclusion criteria for both UI cases and controls comprises: • Bitches recorded as overweight at or before date of neutering. • Bitches with evidence of a pregnancy reported in the EHR.	Same as target trial. Bitches without neuter status recorded or (if evidence that were neutered) without date of neuter documented in the EHR were excluded. A random sampling of eligible individuals, selecting one control per early-onset UI case.
Treatment strategies	(i) Early-age neutering (3 to < 7 months) (ii)Later-age neutering (≥ 7 to ≤ 18 months)	Same as target trial.
Assignment procedures	Eligible bitches are randomly assigned to either age-based strategy of being neutered at the baseline point of 3 months of age. Owners and veterinarians involved in the bitch’s care will be aware of the strategy to which they have been assigned.	Bitches are non-randomly and retrospectively assigned to a treatment strategy. All observed confounding factors will be adjusted to ensure the exchangeability of the groups defined by the initiation of the treatment strategies.
Follow-up period	Follow-up starts at enrolment (3 months of age) and ends at 8.5 years.	Same as target trial.
Censoring	Loss to follow-up, death, or administrative censoring.	Same as target trial.
Outcome	Primary outcome–early-onset UI diagnosis (defined as UI diagnosed at < 8.5 years) (as a binary outcome)	Same as target trial.
Causal contrasts of interest	Per-protocol effect.	Observational analogue of the per-protocol effect.
Estimands	Causal odds for early-onset UI diagnosis at follow-up between arms.	Same as target trial.
Analysis plan	Per-protocol analysis that includes only bitches that complied with the trial protocol. To estimate the per-protocol effect, adjustment for baseline and post-randomisation (time-varying) variables associated with protocol adherence and outcome is needed. Pooled logistic regression modelling is used to evaluate the pooled odds ratio within time intervals. Interaction with follow-up time intervals is additionally evaluated to identify whether the odds vary within the different time intervals.	Same as target trial with adjustment for baseline covariates using inverse probability of treatment weighting. Chronic comorbidities can be considered time-varying, however, due to the time resource to manually collect data and the level of missing data inherent in EHRs, baseline variables only were collected [40]. Censoring due to loss to follow-up or administrative censoring is addressed using inverse probability of censoring weighting.
Adjustment variables	Breed, insurance status, chronic co-morbidities and veterinary group are adjusted for to obtain a principled per protocol estimand (i.e. we assumed that only these variables are simultaneously associated with protocol deviations and outcome).	Breed, insurance status, chronic co-morbidities and vet group. Age at neutering (months) was further adjusted for in the outcome model.

### Descriptive analysis

Demographic data were described. Continuous variables were assessed graphically for their distribution and summarised using median, interquartile range (IQR) and range given most were non-normally distributed. Chi-square testing was used to compare categorical variables and the Student’s t-test or Mann–Whitney U test for univariable comparison of continuous variables between treatment groups as appropriate [41].

### Statistical analysis of the emulated trials

Odds of early-onset UI were compared in bitches neutered later compared to earlier. The statistical methods used in the current study were closely aligned with those used in a recent study emulating a target trial that used a case-control design [31]. The target trial approach has mainly been applied to cohort studies, but can be readily extended to case-control studies when information on treatments or confounders is unavailable or challenging to acquire for the entire cohort but can be obtained for a smaller subset of cases and controls [31, 42].

A causal analysis requires that the four assumptions of consistency, no interference, positivity and no unobserved confounding should hold [26]. The consistency assumption implies that an individual’s potential outcome under their observed exposure is the outcome that will actually be observed for that individual [43] i.e. the exposure specified in the analysis must have enough precision that any variation within the exposure specification would not result in a different outcome [26, 43]. No interference refers to the assumption that the potential outcomes of one individual are unaffected by the treatment assignment of other individuals [44]. Positivity refers to the assumption that the probability of receiving each treatment conditional on measured covariates is greater than zero [26]. Positivity violations occur when certain subgroups (defined by a combination of covariates) in a sample rarely or never receive some treatments of interest [45].

To emulate randomisation at baseline (3 months of age), the following variables were adjusted for to achieve balance between treatment groups: breed, veterinary group, insurance status and chronic comorbidities (as defined and categorised in Table 1 and based on the DAG in Fig 1). The assumption was made that adjusting for these variables were adequate to control for confounding. Age at neutering (months) was included as a continuous covariate in the outcome model to adjust for the differences in age within the two treatment groups.

Inverse probability of treatment weighting (IPTW) was used to evaluate early-onset UI as an outcome. IPTW is a propensity-score based method, with the propensity-score defined as the probability of receiving treatment conditional on observed baseline characteristics [46]. For IPTW, a pseudo-population is created by weighting each individual in the population by the inverse of the conditional probability of receiving the treatment level they indeed received [26]. The goal is to balance covariates between the treatment groups, “up-weighting” and “down-weighting” individuals in each group as necessary [47].

To derive the weights using IPTW, a binary logistic regression model was fitted, with exposure (early or later neutering) as the respective outcome regressed on the confounding variables described above (Table 1). Biologically plausible interaction terms were added to the model and their effect on the standardised mean differences (defined below) were assessed for inclusion, with interaction terms included if they improved covariate balance [48]. The model generated predicted probabilities for each bitch of receiving either treatment (early or later neutering), which were then used to calculate stabilised inverse probability (IP) weights [49]. Extreme weights result when a treated patient has an extremely low or high propensity score, but extreme weights can lead to potentially biased results by increasing the variability of the estimated treatment effect. The use of stabilised weights was achieved by replacing the numerator (which is 1 in the unstabilised weights) with the crude probability of exposure (i.e. given by the propensity score model without covariates) [50, 51].

A pooled logistic regression outcome model (for the presence of early-onset UI including age at neutering as an adjustment variable) was used. In a pooled logistic regression model, the follow-up is split into time intervals from baseline to the end of the study period [52]. The time intervals used in the current study were: ≥ 3 months to < 2.5 years, ≥ 2.5 years to < 4.5 years, ≥ 4.5 years to < 6.5 years and ≥ 6.5 years to < 8.5 years. The method pools observations over all intervals, calculating one causal odds ratio averaged over time [52]. Results from pooled logistic regression analysis have been reported to be close to those from time-dependent covariate Cox regression analysis (using the same data) [52].

Censoring was accounted for in the analysis using inverse probability of censoring weighting (IPCW). IPCW [53] aims to reduce bias introduced by informative censoring (whereby patients are lost to follow-up due to reasons related to the study) [54, 55], with the assumption of conditional exchangeability (concerning the variables used in the censoring model) and correct censoring model specification [56]. IPCW compensates for censored subjects by giving more weight to subjects with similar characteristics that are not censored [57]. To perform IPCW, the probabilities of each bitch being censored within each time interval were calculated using a logistic regression model (with censoring as the outcome regressed on the exposure and confounding variables described). The cumulative probability of a bitch being *uncensored* was calculated and used to determine an IP of censoring weight for each bitch. The final censoring weights were truncated at the 1^st^ and 99^th^ percentile as an additional measure to account for extreme weights [58]. These truncated censoring weights were combined (by multiplication) with the stabilised IP weights generated from IPTW and used to weight each bitch’s contribution to the pooled logistic regression outcome model [26, 31, 53, 55, 59]. An interaction between exposure and time interval was additionally considered in the outcome model, to establish whether the odds ratios for early-onset UI varied within the time intervals. The likelihood ratio test (LRT) was used to determine whether the model including a time interaction fitted significantly better than the model without a time-treatment interaction.

Standardised mean difference (SMD) examined the balance of covariate distribution between exposure groups [60]. For each covariate, SMD between pre- and post-IPTW were calculated, with SMD < 0.1 indicating good covariate balance between the two exposure arms. Effect modification was assessed by adding biologically plausible interaction terms to the outcome models and evaluating their effect on the confidence intervals, Akaike information criterion (AIC) and covariate balance. The weighting procedure means that observations on the same subject will be correlated, therefore a cluster robust variance estimator was used to derive robust standard errors, allowing for clustering [59].

There may be some confounders in a study that are unknown or not measured, and hence unobserved. Sensitivity analysis can examine the extent to which results are affected by values of unmeasured variables [61]. An E-value was computed in the current study, with an E-value defined as the minimum strength of association, on the risk ratio scale, that an unmeasured confounder would need to have with both the treatment and outcome, conditional on the measured covariates, to fully explain away a specific treatment–outcome association. This technique does not require specification of the prevalence of unmeasured confounders and does not make assumptions about their nature [62]. Although the primary outcome was calculated as an odds ratio, and E-values can only be calculated on the risk ratio scale, the odds ratio approximates the risk ratio for rare outcomes (i.e. a prevalence of ≤ 10% in the general population) [63]. An online e-value calculator has been developed and was used, in which the following were specified before calculation: outcome type, estimate type, point estimate, lower and upper confidence intervals, and the true causal effect to which to shift the estimate [62, 64].

Data were checked for internal validity and cleaned in Excel (Microsoft Office Excel 2013, Microsoft Corp.). Analyses were conducted using R version 4.0.2 (R Core Team, Vienna, Austria). The “IPW” package was used to generate IP weights (and validated manually) [65], with code for IPCW derived from Hernán and Robins (2020). Confidence intervals around the primary outcome odds ratio were calculated using bootstrapping [66]. The “halfmoon” package was used to calculate SMDs and “ggplot2” was used to generate a Love Plot [67].

## Results

The study included an underlying VetCompass cohort of 30,953 bitches, born from January 1, 2010, to December 31, 2012, inclusive, neutered between 3 and 18 months, and with at least one EHR before 12 months of age in the VetCompass database. UI search terms yielded 3,728 candidate cases, from which 2,982 (80.0%) were manually reviewed to yield 750 (25.2%) bitches meeting the eligibility criteria for being an early-onset UI case in the emulated trial. Controls were randomly selected from the non-candidate bitches and manually reviewed, on a 1:1 ratio of cases to controls. Therefore, the study included 1,500 bitches overall, of which 612 (40.8%) were neutered early (at 3 to < 7 months) and 888 (59.2%) neutered later (at ≥ 7 to ≤ 18 months).

### Demography of bitches in the emulated trial

The median age at neutering in the “early” group was 6.1 months (IQR 5.7–6.5, range 3.0–6.9), whilst the median age at neutering in the “later” group was 12.0 months (IQR 9.6–14.0, range 7.0–18.0). The most common breeds amongst bitches neutered early were the Labrador Retriever (8.0%; 49), Border Collie (5.4%; 33), Staffordshire Bull Terrier (5.1%; 31) and Jack Russell Terrier (4.7%; 29) in addition to 175 (28.6%) crossbreds. The most common breeds amongst bitches neutered later were the Labrador Retriever (11.1%; 99), Staffordshire Bull Terrier (4.6%; 41), Cocker Spaniel (4.1%; 36), and Boxer (3.3%; 29) in addition to 228 (25.7%) crossbreds (Table 3). Data were 100.0% complete for all covariates, namely breed, chronic comorbidity presence, umbilical hernia, skin disorder, ear disorder, urogenital disorder, insurance status and veterinary group.

**Table 3:** Early neutering (3 to < 7 months) count (%) (n = 612) and later neutering (≥ 7 to ≤ 18 months) count (%) (n = 888) for categorical variables recorded in early-onset UI (< 8.5 years) case and control bitches attending primary-care veterinary practices in the VetCompass™ Programme in the UK. Individual chronic comorbidities were included if at least 8 bitches were affected overall.

Variable	Category	Early neutering No. (%)	Later neutering No. (%)
Breed	Crossbred	175 (28.6)	228 (25.7)
	Purebred—other	182 (29.7)	303 (34.1)
	Labrador Retriever	49 (8.0)	99 (11.1)
	Border Collie	33 (5.4)	25 (2.8)
	Staffordshire Bull Terrier	31 (5.1)	41 (4.6)
	Jack Russell Terrier	29 (4.7)	27 (3.0)
	Cocker Spaniel	25 (4.1)	36 (4.1)
	English Springer Spaniel	23 (3.8)	27 (3.0)
	German Shepherd Dog	18 (2.9)	17 (1.9)
	West Highland White Terrier	17 (2.8)	25 (2.8)
	Shih-tzu	11 (1.8)	16 (1.8)
	Yorkshire Terrier	10 (1.6)	15 (1.7)
	Boxer	9 (1.5)	29 (3.3)
At least one chronic comorbidity	Yes	47 (7.7)	54 (6.1)
	No	565 (92.3)	834 (93.9)
Umbilical hernia	Yes	24 (3.9)	24 (2.7)
	No	588 (96.1)	864 (97.3)
Skin disorder	Yes	7 (1.1)	14 (1.6)
	No	605 (98.9)	874 (98.4)
Ear disorder	Yes	4 (0.7)	4 (0.5)
	No	608 (99.3)	884 (99.5)
Urogenital disorder	Yes	6 (1.0)	2 (0.2)
	No	606 (99.0)	886 (99.8)
Insurance status	Insured	227 (37.1)	354 (39.9)
	Not insured	385 (62.9)	534 (60.1)
Veterinary Group	1	257 (42.0)	320 (36.0)
	2	181 (29.6)	345 (38.9)
	3	98 (16.0)	152 (17.1)
	4	74 (12.1)	67 (7.5)
	5	2 (0.3)	4 (0.5)

### Descriptive analysis of outcome

Descriptive analysis of clinical outcomes reported confounded estimates i.e., before adjusting for confounding. Of the 750 UI cases, 305 (40.7%) were neutered early, whilst 445 (59.3%) were neutered later. Of the 750 controls, 307 (40.9%) were neutered early, whilst 443 (59.1%) were neutered later (p = 0.958). The median time (years) to early-onset UI diagnosis in bitches neutered early (2.7 years, IQR 1.2–5.0, range 0.1–8.0) did not significantly differ to bitches neutered later (2.7 years, IQR 1.5–4.6, range 0.1–7.7) (p = 0.955).

### Model evaluation

**Fig 2:**
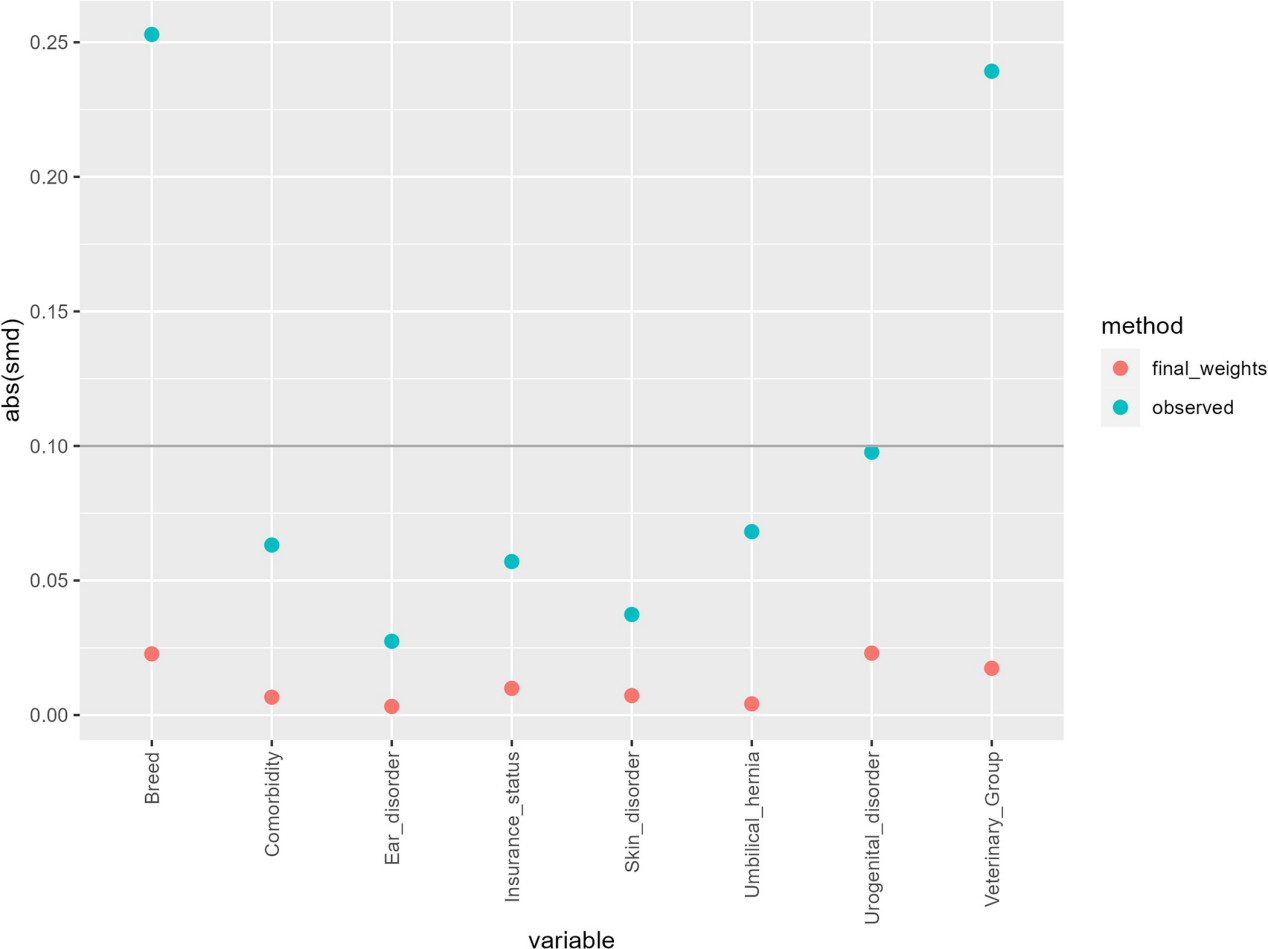
Standardised mean differences (SMD) before and after applying inverse probability weighting. The “observed” method represents the absolute SMD of the prespecified covariates pre-weighting and the “final_weights” the absolute SMD post-weighting.

The standardised mean differences (SMDs) between groups for each of the covariates pre- and post-IPTW are shown in Fig 2. The SMDs in the weighted sample were below 0.1 for all covariates, indicating well-balanced groups post-weighting (Fig 2).

The median propensity score in bitches neutered early was 0.57 (range 0.13–0.83), whilst the median propensity score in bitches neutered later was 0.60 (range 0.19–1.00). The range of IP weights (when treatment weights were combined with truncated censoring weights) was 0.30 to 5.60. The inclusion of an interaction term between breed and chronic comorbidity improved covariate balance (based on SMDs). Therefore, the final model included the following covariates to generate propensity scores: breed, chronic comorbidity, breed*chronic comorbidity, umbilical hernia, skin disorder, ear disorder, urogenital disorder, insurance status and veterinary group.

### Emulated trial results

After adjusting for confounding between early and later neutering groups using IPTW, and accounting for censoring using IPCW, later neutering reduced the risk of early-onset UI compared with early neutering. Specifically, the causal odds ratio in bitches neutered later versus early was 0.80 (95% CI 0.54 to 0.97) i.e., the odds of developing early-onset UI are 20% lower in bitches neutered later compared to bitches neutered early.

Treatment interaction with follow-up time intervals in the outcome model was explored to establish whether the odds for early-onset UI varied over time. However, there was no evidence that the model with interactions fitted the data significantly better (LRT p-value < 0.001) and therefore the model without a time interaction term was reported.

An E-value of 1.81 (lower CI 1.21) was calculated to assess the risk ratio required for an unmeasured confounder (given the measured covariates) to fully explain away the treatment–outcome association.

## Discussion

Target trial emulation studies are increasing within the field of human epidemiology [68], with studies beginning to adopt this approach for veterinary observational data [27, 28]. The target trial emulation framework aims for clarity when specifying the ‘target trial’, minimising common design and methodological flaws often evident in the analysis of non-randomised studies, with each step transparent and accessible [25, 27, 28]. Previous observational studies using VetCompass data have explored the effect of age at neutering on early-onset UI diagnosis in bitches but these were limited to reporting associations rather than causal effects [3, 12]. The current study used causal inference methodology to overcome this limitation by aiming to determine cause rather than just reporting association. The current study also provides an example of how this target trial emulation approach can be applied to veterinary EHR data in the case-control setting.

The current study limited UI to early-onset cases (i.e., diagnosed before 8.5 years of age) because older or geriatric bitches may have comorbidities resulting in micturition disorders [69] and degenerative changes that may be more age-related and less influenced by neuter status and the neutering choices that owners and veterinary surgeons make for bitches in early life [3]. Studies including all UI cases could therefore represent a wide variety of other causes for UI, such as age degeneration, that are less amenable to change by human intervention than the chosen age for neutering, and therefore evaluating early-onset UI allowed for more specific phenotype selection [3, 12].

After adjusting for confounding in the early neutered and later neutered groups using IPTW, and accounting for censoring using IPCW, bitches neutered later had 20% lower odds (95% CI 3% to 46%) of early-onset UI diagnosis compared with bitches neutered early. Although neutering itself has consistently been reported as an important risk factor for UI diagnosis in bitches [3, 8, 11, 12, 16, 17], until now, the literature has offered much less evidence about the effects of age on neutering. To date, there has been some limited evidence that neutering < 7 months increases the risk of UI in bitches [3, 18, 19], in line with the current study findings. That said, the effect for neuter itself (as a binary variable) appears stronger than the effect for age at neutering, with neutered bitches under primary-care in the UK at 3.01 times the odds of UI compared with entire bitches in a previous case-control study [12].

The current study identified an overall reduced odds of early-onset UI diagnosis in bitches neutered later (compared with early). A previous observational study based on UK primary-care data identified bitches neutered before 6 months of age at increased hazard of early-onset UI diagnosis within the first 2 years following neutering [12], although this effect was not consistent across the entire follow-up period. A greater number of UI cases were included in the current study compared with the aforementioned study, which may in part account for the differences due to statistical power, however, implementation of the target trial framework provides a more accurate representation of a true causal effect [25]. The current study was built around one specific research question, with confounders derived from a DAG. Conversely, the previous observational study potentially could promote “Table 2 Fallacy” whereby presenting multiple effect measures estimated from the same model encourages the reader to interpret each estimate as standalone results. However, the interpretation of a confounder effect estimate may be different than for the exposure effect estimate, due to underlying differences in the causal model [27, 28, 70].

UI in bitches kept as family pets has been reported to cause disharmony in 10 to 20% of affected households. Owners have described feelings of anger and frustration [15], even contemplating euthanasia of the affected animal in certain circumstances [8, 71]. Additionally, UI can directly impact the welfare of the bitch by increasing the risk of urinary tract infections and urine scald [29]. The majority of bitches with UI are clinically managed with medical therapy [22], most commonly phenylpropanolamine (PPA) [72, 73], with response rates from 85.7% to 97% and minimal side effects reported [72, 74–76]. However, UI is generally permanent from diagnosis, with lifelong medication imposing a significant financial impact [22]. Consequently, due to the possible impact on the owner-animal bond, the welfare concerns for the bitch, the financial implications of treatment, as well as the significance of UI in the decision to neuter [3, 12], the current study findings are noteworthy by identifying that a delay in the age at neutering could prevent a significant number of bitches from developing early-onset UI. However, UI risk is one feature of a tailored decision for each dog, and there might be alternative justifications for early neutering, for example in the charity setting as a means of population control.

The limitations of this study are largely based on the nature of retrospective analysis of electronic health record data, including issues related to unobserved confounding, missing and misclassified data and application of a case definition to the data available [27, 28, 77]. Confounders were collected at baseline, but there might be time-varying confounders that influence outcome post-baseline. Of note, obesity is believed to worsen the degree of UI [78]. Although bitches overweight or obese before neutering were excluded, data on overweight status post-neutering was not collected. Underreporting of overweight status in veterinary EHRs is well-recognised [79], therefore there is a time-data trade-off between the time resource required to manually collect data and the reliability and quality of the expected data.

Age at neutering is closely linked with neutering relative to first oestrus [17]. However, information on the date of first oestrus, either in absolute terms or in relation to the timing of neutering, was infrequently reported in the primary-care EHR data [12]. Therefore, the current study focused on temporal age at neutering as the exposure. Previous evidence has suggested 8.7% bitches have had or started their first oestrus by 7 months of age, therefore the majority of bitches neutered < 7 months will not have had a season [20]. However, the oestrus status in bitches neutered ≥ 7 to ≤ 18 months is less certain, thereby limiting inference from the current study about the effect of neutering relative to first oestrus on early-onset UI diagnosis. Likewise, the current study did not evaluate the surgical method of neutering due to the limited availability of this information from the clinical records [12]. There is currently no evidence for a significant association between the type of surgery performed (namely ovariectomy and ovariohysterectomy) and occurrence of UI [80–86].

Time zero was set at 3 months to allow equivalent follow-up between the early and later neutering groups, and to align more closely with a target trial. Although the early neutering group had the scope for a longer follow-up period post-neutering, the median time to early-onset UI diagnosis was the same in bitches neutered early and bitches neutered later (2.7 years). Additionally, the median time from neutering to UI has previously been reported to range between 1.9–5.0 years [3, 11, 15, 69, 87, 88], thus the follow-up specified in the current study should allow for acceptable consistency between treatment groups in capturing cases.

Expert advice was enlisted in constructing the DAG; however, there remains a possibility that unmeasured confounding might impact the calculated odds ratios. To assess this, an E-value was computed to measure the minimum level of association, expressed as a risk ratio, that an unmeasured confounder would need to have with both the exposure and outcome, considering the measured covariates, in order to fully explain away the treatment–outcome effect [27, 28, 62]. The E-value was 1.81, with the lower estimate 1.21, suggesting that the odds ratio for early-onset UI diagnosis of 0.80 could be explained away by an unmeasured confounder that is associated with both exposure (age at neutering) and outcome (early-onset UI diagnosis) by a risk ratio of 1.81 [62]. E-values should be interpreted in the broader context and with other strengths and weaknesses of the study and design [27, 28, 62], but these results suggest that relatively weak unmeasured confounding could alter the causal interpretation. Whilst the results of the current study suggest age at neutering within those bitches that are neutered is an important contributor to the development of UI, the effect of neutering overall still appears to be stronger.

## Conclusions

Using causal inference methodology, the study findings show that later-age neutering (≥ 7 to ≤ 18 months) causes a 20% reduction (95% CI 3% to 46%) in the odds of early-onset UI diagnosis compared with early-age neutering (3 to < 7 months). Although a decision to neuter a bitch is based on many other factors as well as UI risk, these results suggest that clinical decision-making to undertake early-age neutering (< 7 months) should be carefully considered and be well justified.

## Abbreviations

AIC: Akaike information criterion
CI: confidence interval
DAG: directed acyclic graph
EHR: electronic health record
IP: inverse probability
IPTW: inverse probability of treatment weighting
IQR: interquartile range
OR: odds ratio
RCT: randomised controlled trial
RD: risk difference
SMD: standardised mean difference
UI: urinary incontinence
